# Characterizing the Blood Oxygen Level-Dependent Fluctuations in Musculoskeletal Tumours Using Functional Magnetic Resonance Imaging

**DOI:** 10.1038/srep36522

**Published:** 2016-11-15

**Authors:** Li-Sha Duan, Meng-Jun Wang, Feng Sun, Zhen-Jiang Zhao, Mei Xing, Yu-Feng Zang, Steven Louis, Sheng-Jie Cui, Jian-Ling Cui, Han Zhang

**Affiliations:** 1 Department of Radiology, The Third Hospital of Hebei Medical University, Shijiazhuang, Hebei 050051, China; 2 Hebei Province Biomechanical Key Laboratory of Orthopedics, Shijiazhuang, Hebei 050051, China; 3 Center for Cognition and Brain Disorders, Hangzhou Normal University, Hangzhou, Zhejiang 311121, China; 4 Zhejiang Key Laboratory for Research in Assessment of Cognitive Impairments, Hangzhou, Zhejiang 310015, China; 5 Physics Department, Oakland University, 190 Science and Engineering Building, 2200 N. Squirrel Road, Rochester, Michigan 48309-4401, USA; 6 Department of Anatomy and Cell Biology, Wayne State University School of Medicine, 540 East Canfield Avenue, Detroit, Michigan 48201, USA; 7 Department of Radiology and BRIC, University of North Carolina at Chapel Hill, NC 27599, USA

## Abstract

This study characterized the blood oxygen level-dependent (BOLD) fluctuations in benign and malignant musculoskeletal tumours via power spectrum analyses in pre-established low-frequency bands. BOLD MRI and T1-weighted imaging (T1WI) were collected for 52 patients with musculoskeletal tumours. Three ROIs were drawn on the T1WI image in the tumours’ central regions, peripheral regions and neighbouring tissue. The power spectrum of the BOLD within each ROI was calculated and divided into the following four frequency bands: 0.01–0.027 Hz, 0.027–0.073 Hz, 0.073–0.198 Hz, and 0.198–0.25 Hz. ANOVA was conducted for each frequency band with the following two factors: the location of the region of interest (LoR, three levels: tumour “centre”, “peripheral” and “healthy tissue”) and tumour characteristic (TC, two levels: “malignant” and “benign”). There was a significant main effect of LoR in the frequencies of 0.073–0.198 Hz and 0.198–0.25 Hz. These data were further processed with post-hoc pair-wise comparisons. BOLD fluctuations at 0.073–0.198 Hz were stronger in the peripheral than central regions of the malignant tumours; however, no such difference was observed for the benign tumours. Our findings provide evidence that the BOLD signal fluctuates with spatial heterogeneity in malignant musculoskeletal tumours at the frequency band of 0.073–0.198 Hz.

Acute hypoxia (i.e., transient cycles of hypoxia–reoxygenation) is known to occur in solid tumours^1^ and may be associated with resistance to radiation therapy^2^,^3^, impaired delivery of chemotherapeutic agents^4^ or metastasis development^5^. The origin of acute hypoxia is not firmly established; however, it is generally believed to result from tumour blood flow instabilities. Flow fluctuations may result from arteriolar vasomotion^6^,^7^, haemodynamic changes resulting from a disorganized vascular hierarchy^8^, and other factors.

Several methods can detect acute hypoxia. Oxygen microelectrodes^9^,^10^ and fibre-optic oxygen-sensing device^11^,^12^ can invasively detect the spontaneous oxygen tension fluctuations in animal tumour models by inserting a microprobe into the tumour. In addition to their invasive nature, another drawback of these techniques is the restriction of spatial resolution. Additionally, although phosphorescence lifetime imaging to detect the partial oxygen tension through a cutaneous window chamber has high spatial resolution, it is an invasive method and is not allowed in humans^13^. Additionally, hypoxia can be detected using positron emission tomography (PET) with a hypoxia-specific radio-tracer. Acute hypoxia may be identified through acute changes in the intensity of the tracer in human tumours with a mathematical model^14^. However, detection of acute hypoxia using PET requires several injections of radiotracers over days. Moreover, in intratumoural injections of perfluorocarbon (PFC) compounds, the relaxation rate 1/T_1_ is proportional to the percentage of O_2_ acquired. This is also an invasive procedure and has limited temporal resolution^15^.

Blood oxygen level-dependent (BOLD) functional MRI that depends on deoxyhaemoglobin as an endogenous contrast agent is sensitive to changes in blood flow and deoxyhaemoglobin content with high temporal and spatial resolution^16^,^17^,^18^. The spatial and temporal heterogeneity of spontaneous T2* (BOLD) MR signal fluctuations was first observed in an implanted fibrosarcoma mouse model by Baudelet *et al.*^19^,^20^ and later confirmed in tumour xenograft models of colorectal carcinoma by Goncalves and colleagues^21^.

However, the occurrence of spontaneous BOLD signal fluctuations in the tumours described above has never been demonstrated in human tumours. We examined patients with malignant or benign musculoskeletal (MSK) tumours using BOLD for 6 minutes per patient and analysed the BOLD fluctuation characteristic in the centre and periphery of each tumour via power spectrum analyses. The aim of this study was to assess the spatial heterogeneity of the BOLD signal fluctuations in human MSK tumours via power spectrum analyses in pre-established low-frequency bands.

## Results

### Participants and Histopathology

Of the 52 MSK tumour patients, four had excessive body motions (translation >3 mm or rotation >3°) that could have introduced severe artefacts in BOLD signals; these patients were removed from further analyses. The data from the remaining 48 patients were entered into further analyses (Table 1). Histological results showed that 35 patients had malignant tumours; the others^13^ had benign tumours. The malignant MSK tumours included osteosarcoma (12 cases), malignant fibrous histiocytoma^4^, synovial sarcoma^3^, two cases each with alveolar sarcoma, Ewing sarcoma, liposarcoma, metastatic renal clear cell carcinoma and chondrosarcoma, and one case each with metastatic adenocarcinoma, primitive neuroectodermal tumour, malignant chondroblastoma, epidermoid leiomyosarcoma, fibrosarcoma and malignant tenosynovial giant cell tumour. The benign MSK tumours consisted of giant cell tumours of bone in 10 cases and one case each with neurofibroma, lipoma and fibromatosis.

### Variability in BOLD Fluctuations Within and Around MSK tumours

**Figure 1:**
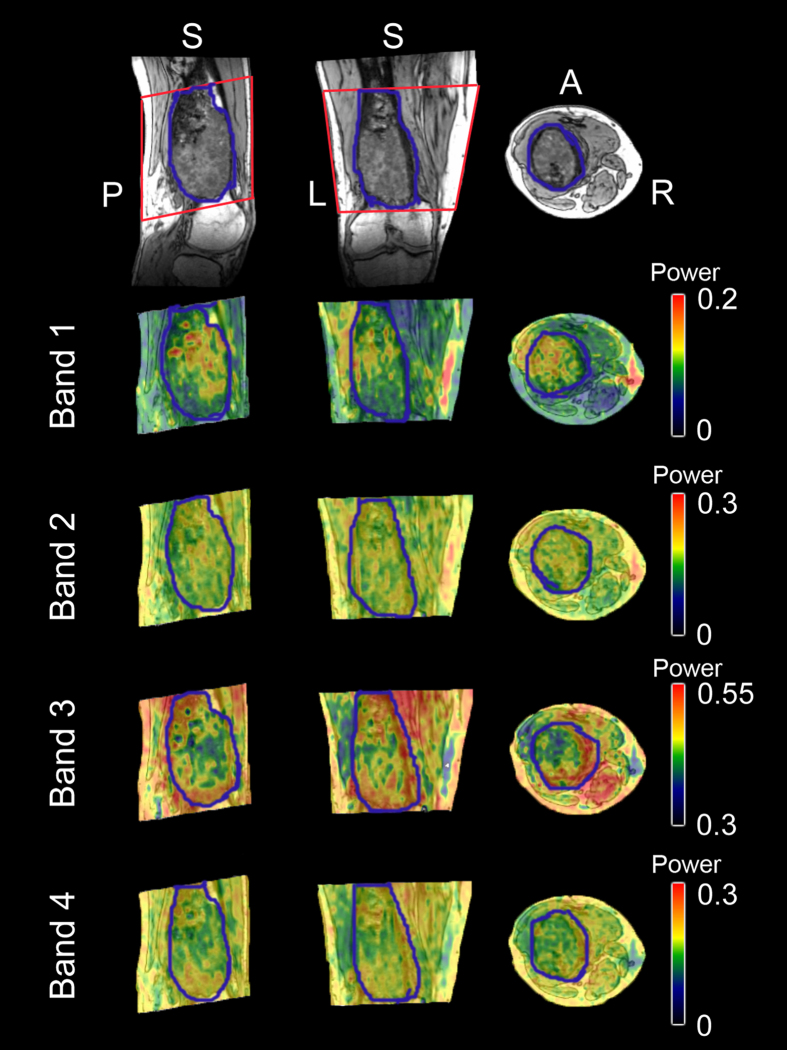
BOLD fluctuation magnitude within and around a malignant MSK tumour from a randomly selected patient. The first row shows the 3DT1-weighted image of the MSK tumour in the femur and the BOLD-fMRI field-of-view (within the red rectangle). The second to the last row shows the BOLD fluctuation magnitude (normalized power) within frequency bands 1–4, which were rendered onto the T1-weighted image.

There was a difference in BOLD fluctuations (measured according to the averaged normalized power in each frequency band) between various locations within and around the MSK tumour tissue. Images derived from a voxel-wise calculation of the BOLD fluctuation magnitude for various frequency bands from a randomly selected patient are shown in Fig. 1. As can be observed, there is a visible variability in the BOLD fluctuation power of BOLD signal within and around this malignant MSK tumour.

### Statistical Analyses

The power spectrum of the BOLD fMRI signal was divided into the following four frequency bands according to a previous study^22^: band-1, 0.01–0.027 Hz; band-2, 0.027–0.073 Hz; band-3, 0.073–0.198 Hz; and band-4, 0.198–0.25 Hz. The reason for adopting these cut-offs is that the BOLD fluctuation power within the four bands was approximately equal^22^. These results are statistically shown in our 2-by-3 ANOVA analysis, which used tumour characteristic (TC, consisting of two levels: “malignant” and “benign”) and the location of ROI (LoR, consisting of three levels: tumour “centre/c” and “peripheral/p”, and “healthy tissue”) as two factors. As shown in Table 2, there was no significant main effect for the TC or the interaction effect between TC and LoR in all frequency bands. Notably, Table 2 also showed a significant main effect for LoR in the frequency of band-3 (0.073–0.198 Hz) (*P* = 0.0167) and band-4 (0.198–0.25 Hz) (*P* = 0.0243).

**Figure 2:**
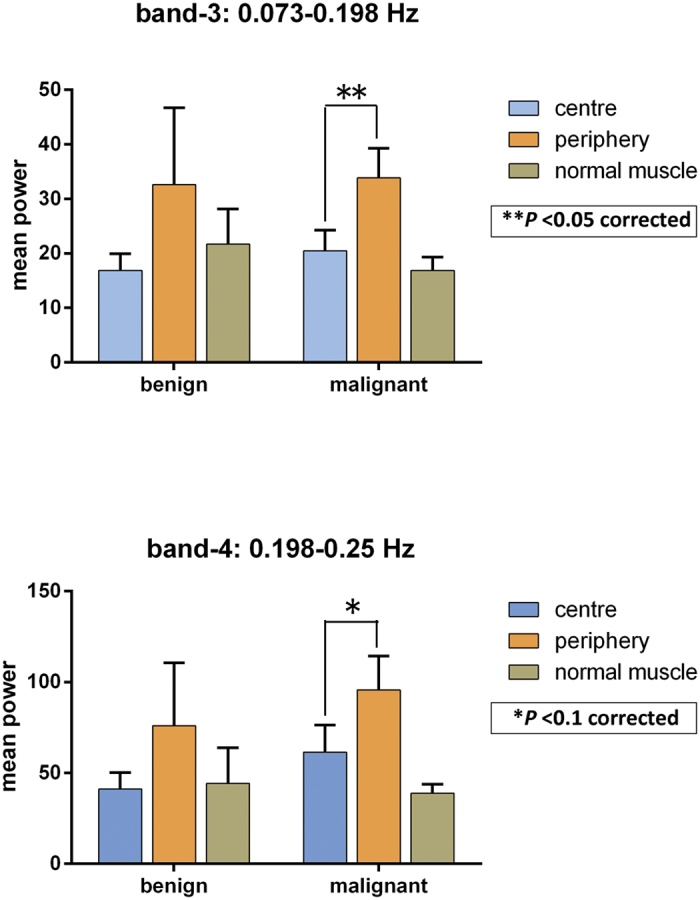
Post-hoc pairwise comparison analysis with Sidak adjustment in 2 frequency bands between benign and malignant tumours. **P < 0.05 after correction; *P < 0.1 after correction.

After the ANOVA analyses, the data with a significant main effect of LoR was further processed with post-hoc pair-wise comparison, as shown in Table 3. For malignant tumours, there was a statistically significant difference (*P* < 0.05, corrected) in BOLD fluctuation power in band-3 and a trend towards a significant difference (*P* < 0.1, corrected) in band-4 between the tumour periphery and the centre (i.e., p > c); the benign tumours showed no such difference (Fig. 2).

### Intra- and Inter-rater Reliability in ROI definition

To determine whether these results were affected by the ROI selection, the intra- and inter-rater reliability of the BOLD fluctuation power between ROI selections were calculated. The intra-rater value ICC for the two measurements performed by the primary radiologist (M.J.W) were 0.67 ± 0.13 (range: 0.49–0.93), which means that the reliability of the ROI definition is fair to excellent (Table 4). The inter-rater ICC values between the ROI definitions of radiologists M.J.W and Z.J.Z also indicated that the reliability is fair to excellent, which were 0.62 ± 0.15 (range: 0.37–0.81, between Z.J.Z’s measurement and M.J.W’s first measurement) and 0.71 ± 0.11 (range: 0.54–0.87, between Z.J.Z’s measurement and M.J.W’s second measurement)(Table 4).

## Discussion

This is the first time that human MSK tumours have been characterized using a frequency spectrum analysis of BOLD in specific frequency bands. Taking our observations together, ANOVA showed that in the frequency bands 0.073–0.198 Hz (band-3, *P* = 0.0167) and 0.198–0.25 Hz (band-4, *P* = 0.0243), there was a significant difference in the BOLD fluctuation power between the centre and the periphery of the MSK tumour and the surrounding normal muscle (Table 2). The post-hoc simple effect analysis showed that only in the malignant tumour were there significantly (band-3, *P* < 0.05, corrected) or a trend towards significantly (band-4, *P* < 0.1, corrected) higher BOLD fluctuations in the peripheral tumour region than the central tumour region; there was no such difference for benign MSK tumours (Table 3).

According to Ogawa’s group, the source of BOLD fMRI signal displays the ratio of oxyhaemoglobin and deoxygenated haemoglobin in the arterioles, capillaries and post-capillary venules^23^. The BOLD signal has also been shown to be positively related to blood flow or the partial pressure of oxygen in normal tissue or tumour tissue^20^,^24^. Flow fluctuation is also called flowmotion. Flowmotion as detected via perfusion imaging is closely associated with the BOLD fluctuation^25^,^26^. Blood flow fluctuation may induce acute hypoxia in tumours. Thus, spontaneous BOLD fluctuations are likely related to acute hypoxia in tumours^18^,^19^,^20^,^21^.

The BOLD fluctuations in band-3 in the periphery of the malignant MSK tumours were larger than those in the centre in our series, whereas for benign tumours there was no significant difference in all frequency bands between the centre and periphery. Häfner detected the perfusion of human cutaneous malignant melanomas, benign melanocytic nevi and normal skin^27^. The wavelet analysis reveals that the mean vasomotion scale variance values obtained from healthy skin significantly differed from the margins and centres of cutaneous malignant melanomas but did not significantly differ between the margin and centre of benign melanocytic nevi. These findings support our results. However, the amplitude difference of BOLD fluctuation between the centre and periphery in malignant MSK tumours was only at the band of 0.073–0.198 Hz; the cause for this remains to be determined.

Interestingly, the frequency band of 0.073–0.198 Hz in tumours has been suggested to be caused primarily by vascular myogenic activity in vasomotion^28^,^29^. Further study is needed to determine whether this frequency band detected via BOLD has the same implications.

The frequency of oxygen fluctuation (hypoxia-reoxygenation) detected via microprobe^9^,^11^,^12^ or ^19^F MRI methods^9^ ranges widely from 0.00035–0.0167 Hz, with an average of approximately 0.001 Hz.

Our MR scanning protocol precluded the analysis of frequency bands higher than 0.25 Hz (due to the limited temporal sampling rate, 2 s) and less than 0.00278 Hz (due to the 6-minute signal acquisition time). To elucidate the spatial heterogeneity of the amplitude of the BOLD signal at some fluctuation frequenceses, we tested the differences between the benign and malignant MSK tumours as well as between the centre and periphery of the MSK tumours. Only an amplitude of 0.073–0.198 Hz was significantly higher for the peripheral tumour region in the malignant tumour. The significant frequency band (0.073–0.198 Hz) detected in our study may provide new pathological and physiological significance in characterizing the acute hypoxia fluctuations in the tumours.

In the present study, 48 patients with malignant or benign MSK tumours were studied; this represents a relatively small sample size. The substantially smaller sample size of benign tumours compared with malignant tumours may induce statistical bias.

In conclusion, we found that in the frequency band of 0.073–0.198 Hz, the fMRI BOLD fluctuation power of the central is lower than the peripheral regions in human malignant MSK tumours; however, no significant difference was found for benign tumours. The meaning of this difference remains under investigation.

## Materials and Methods

### Participants

Fifty-two patients (34 males and 18 females; age 39.9 ± 17.8 years, 14–75 years) who were hospitalized consecutively from February 2009 to June 2011 with primary MSK tumours were included in this study. All patients were drug-naive and without any treatment prior to MR imaging. The demographic and clinical information are summarized in Table 1. The inclusion criteria were the following: (1) the tumour had to be predominately solid; (2) the tumour dimension had to be larger than 3 cm in each of the axial, sagittal and coronal plains to facilitate ROI drawing; and (3) the tumour had no extensive intra tumour necrosis or bleeding. Tumour characteristics were determined via histological testing after resection or needle biopsy. This study was approved by the institutional research ethics board in the Third Hospital of Hebei Medical University, China. All experiments were performed in accordance with relevant guidelines and regulations, and all patients signed written informed consent before the study was carried out.

### MR Imaging

MR imaging was performed on a 1.5 T Siemens MR scanning system (Avanto, Siemens, Erlangen, Germany). Scanning was performed with patients lying in a supine position and being asked to relax throughout the scan. Depending on which body part had tumours and was scanned, image acquisition used one of the following coils: a large circularly polarized flexible coil, an eight-channel knee coil or an eight-channel body array coil. The scanning protocol included three-dimensional turbo fast low angled shot (FLASH) T1-weighted images (3D T1WI) (repetition time/echo time, 1900/2.97 ms; flip angle, 15°; number of slices, 176; slice thickness/gap, 1/0.5 mm; acquisition matrix, 256 × 246; field of view, 220 mm × 220 mm). Two-dimensional echo planar imaging (EPI) BOLD fMRI with 20 axial slices was also taken (repetition time/echo time, 2000/40 ms; slice thickness/gap, 5/1 mm; field of view, 220 mm × 220 mm; acquisition matrix, 64 × 64; voxel size, 3.44 mm × 3.44 mm × 6.0 mm; number of dummy scans, 3; scanning time, 6 min, 177 frames total). 3DT1WI images were taken to identify the tumour locations; the BOLD fMRI scan was taken to detect BOLD fluctuations in corresponding body tissues.

### Data Preprocessing

**Figure 3:**
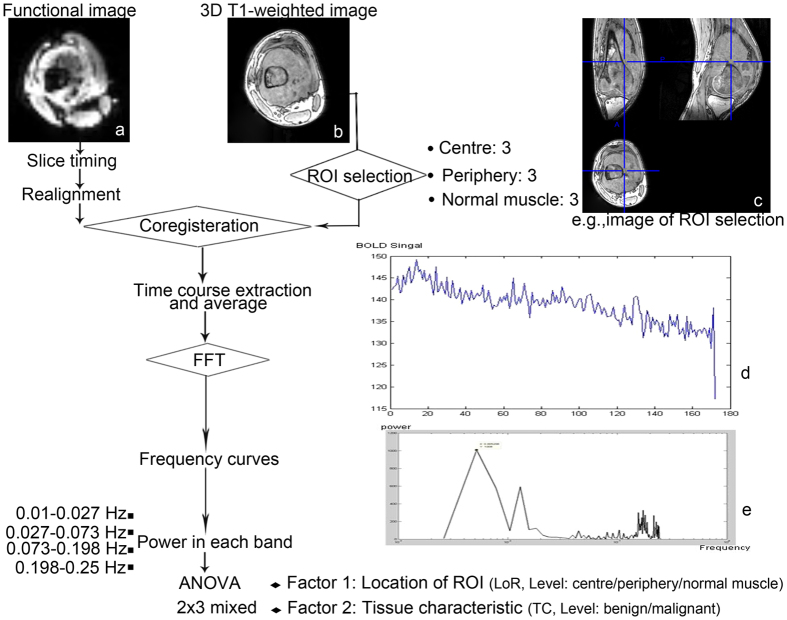
Schematic illustration of the course to data processing and analysis. The selected patient is a 19-year-old man with osteosarcoma in his left femur. (**a**) Functional image. (**b**) 3D T1-weighted image (**c**) A ROI in the centre of the tumour with a 4-mm radius. Its centre coordinate is (97,114,132). (**d**) Time course after preprocessing. (**e**) Power spectrum using FFT. ROI = region of interest; FFT = fast Fourier transformation; ANOVA = analysis of variance.

The fMRI data were preprocessed using DPARSFA v2.4^30^ and REST v1.8^31^ based on SPM8 (www.fil.ion.ucl.ac.uk/spm) and Matlab 2010a (MathWorks, Inc., Natick, MA, USA). See Fig. 3 for a flowchart of the entire analysis procedure. The preprocessing steps included the following: (1) the fMRI data were converted to Neuroimaging Informatics Technology Initiative (NIfTI) format; (2) the first five frames of each patient’s fMRI data were discarded to allow for MR machine equilibrium and the patients’ adaption; (3) within-frame slice acquisition timing was corrected; (4) patient’s body motion was corrected using a 6-parameter rigid-body transformation, and the patients found to have excessive body motion (>3 mm or >3 degrees) were excluded from further analyses; (5) the three-dimensional T1 image of each patient was co-registered to the patient’s own averaged BOLD fMRI image to spatially match the two modalities. Note that we did not perform spatial re-sampling or smoothing because we intended to keep the original BOLD signal and to minimize spatial blurring influence and signal contamination caused by neighbouring voxels.

### ROI Drawing

BOLD fMRI images provide critical information about blood flow and oxygen levels but do not readily allow visual distinction between tumour and non-tumour regions due to the limited spatial resolution. Therefore, we defined ROIs based on 3D T1WI and then transformed the coordinates of the ROIs to match the BOLD fMRI images according to the projection derived from the co-registration. Prior to ROI definition, the tumour tissue was divided into three nearly concentric regions (centre, transition and periphery regions) from the tumour centre to periphery as equally as possible by an experienced radiologist (M.J.W) according to previous studies^32^. As the transition area within a tumour was difficult to be differentiated from the tumour centre and peripheral regions^29^, we intended not to use it as an ROI. Within tumours, ROIs were only chosen from the tumour centre and periphery. The normal muscle surrounding the tumour but without abnormal signal was also defined as one ROI type. Therefore nine ROIs, three in the centre (c), three in the periphery (p) of the tumour and three in the surrounding normal muscle (m), with a radius of 4 mm each, were chosen for each patient. The three ROIs belonging to the same tissue type (c, p, or m) were chosen to spread as far as possible^32^ from each other to avoid systemic noise contamination. The regions with necrosis, calcification or that included large arteries or veins were avoided when defining the ROIs. For ten patients, the fMRI data did not include adequate normal muscle tissue because of the limited imaging field of view; we did not define the ROIs of the normal muscle for these patient (i.e., the muscle ROIs were available for 38 of 48 patients). Each ROI contained five voxels of the BOLD fMRI.

### Power Spectrum Analyses

For each patient, averaged BOLD time series across the five voxels in each ROI were calculated, and a linear trend was removed from them. The averaged BOLD signal for each ROI was transformed from the temporal to frequency domain with a fast Fourier transformation using Matlab (i.e., frequency power spectrum in which the “power” is the square of the BOLD fluctuation amplitude, Fig. 1). This power spectrum was then divided into the following four frequency bands according to a previous study^17^: band-1, 0.01–0.027 Hz; band-2, 0.027–0.073 Hz; band-3, 0.073–0.198 Hz; and band-4, 0.198–0.25 Hz. The power values for each ROI were then normalized by dividing the total power value of the entire frequency band (0–0.25 Hz) to facilitate inter-patient comparisons (similar to the calculation of fractional amplitude of low-frequency fluctuation in Zang *et al.*^33^). The normalized power values for the three ROIs belonging to the same tissue type were further averaged for each frequency band for the Analysis of Variance (ANOVA).

### Statistical Analyses

We investigated whether there was significant difference in BOLD fluctuation power between different locations of ROIs or between malignant and benign tumours. Specifically, there were two main factors in the ANOVA model as follows: (1) tumour characteristics (TC), which consisted of two levels (i.e., either benign or malignant tumour) and (2) the location of the ROI (LoR), which has three levels ((i.e., the tumour centre (c), the tumour periphery (p), and the normal muscle (m)). The 2 × 3 mixed-designed ANOVA (the two fixed effect factors stated above and one random effect factor, “subject”) was performed on the averaged BOLD fluctuation power values on each frequency band. Two main effects (i.e., the TC and LoR main effects) and one interaction effect (i.e., TC-by-LoR) were assessed. For any significant (*P* < 0.05, uncorrected) main effect derived from the ANOVA in any frequency band, simple effect analyses using post-hoc pair-wise comparisons were conducted to further explore the direction of the difference in BOLD fluctuation power. The significance level was set to *P* < 0.05 and corrected for multiple comparisons with Sidak adjustment.

### Intra-Rater and Inter-Rater Reliability Assessment

We chose relatively objective ROI selection criteria (described above) to reduce the introduction of errors due to human subjectivity. To assess the intra-rater reliability of our ROI selection criteria, we performed the ROI definition twice with our primary radiologist (M.J.W.). The first and second round of ROI definition were separated by one week according to the same criteria but fully independent from each other. To determine inter-rater reliability, all of the ROIs were also independently drawn by a second experienced radiologist (Z.J.Z.) who was blinded to all previous results. The averaged BOLD fluctuation power value for each tissue type for each patient was calculated and fed into intra-class correlation coefficients (ICC) calculation. We calculated the intra-rater and inter-rater ICCs for each of the frequency bands. All ICC values were evaluated with the criterion reported in Zhang *et al.*^34^. An ICC value larger than 0.75 indicates “excellent” reliability, between 0.59–0.75 indicates “good”, 0.40–0.58 indicates “fair”, and below 0.40 indicates “poor” reliability.

## Additional Information

**How to cite this article**: Duan, L.-S. *et al.* Characterizing the Blood Oxygen Level-Dependent Fluctuations in Musculoskeletal Tumours Using Functional Magnetic Resonance Imaging. *Sci. Rep.* **6**, 36522; doi: 10.1038/srep36522 (2016).

**Publisher’s note**: Springer Nature remains neutral with regard to jurisdictional claims in published maps and institutional affiliations.

## Figures and Tables

**Table 1:** Demographic and Tumour Information of 48 Cases.

Variables (malignant)^a^	Values	Variables (benign)^b^	Values
**Cases**	35	**Cases**	13
**Age (yrs)**		**Age (yrs)**	
Mean ± sd	35.74 ± 17.97	Mean ± sd	41.62 ± 16.18
Range	14–75	Range	18–70
**Males-no. (%)**	24 (68.60)	**Males-no. (%)**	7 (53.80)
**Position-no. (%)**		**Position-no. (%)**	
Femur	13 (37.14)	Femur	4 (30.77)
Tibia	6 (17.14)	Tibia	5 (38.46)
Fibula	2 (5.71)	Fibula	1 (7.70)
Humerus	2 (5.71)	Humerus	n.s.
Sacrum	1 (2.86)	Sacrum	n.s.
Os Innominatum	1 (2.86)	Os Innominatum	n.s.
Calcaneus	1 (2.86)	Calcaneus	n.s.
Soft tissue	9 (25.71)	Soft tissue	3 (23.08)
Tumour volume *(cm^**3**^)		Tumour volume *(cm^**3**^)	
Mean ± sd	163.02 ± 140.88	Mean ± sd	159.13 ± 180.96
Range	23.40–546.60	Range	19.60–588.60

^a^Osteosarcoma, malignant fibrous histiocytoma, synovial sarcoma, alveolar sarcoma, Ewing sarcoma, liposarcoma, chondrosarcoma, metastatic adenocarcinoma, metastatic renal clear cell carcinoma, primitive neuroectodermal tumour, malignant chondroblastoma, epidermoid leiomyosarcoma, fibrosarcoma, malignant tenosynovial giant cell tumour.

^b^Giant cell tumour of bone, neurofibroma, lipoma, fibromatosis. **yrs** = years; **no**. = number. *Selected 37 tumours to calculate their volumes using the formula (4/3) π abc (a, b, c = the 3 radii).

**Table 2:** The ANOVA Table of Power Difference in 48 Cases.

	Freq band (Hz)	SS	MS	*F* value**	*P* value
Main effect of LoR	Band-1: 0.01–0.027	4516.10	2258.06	2.73	0.0714
	Band-2: 0.027–0.073	2487.50	1243.77	2.92	0.0596
	Band-3: 0.073–0.198	4971.00	2485.51	4.30	0.0167*
	Band-4: 0.198–0.25	41110.30	20555.10	3.89	0.0243*
Main effect of TC	Band-1: 0.01–0.027	461.60	461.58	0.26	0.6117
	Band-2: 0.027–0.073	501.70	501.69	0.68	0.4145
	Band-3: 0.073–0.198	0.40	0.39	0	0.9846
	Band-4: 0.198–0.25	2959.20	2959.20	0.26	0.6156
Interaction effect of LoR and TC	Band-1: 0.01–0.027	1527.20	763.59	0.92	0.4018
	Band-2: 0.027–0.073	9.90	4.94	0.01	0.9885
	Band-3: 0.073–0.198	291.00	145.51	0.25	0.7780
	Band-4: 0.198–0.25	4607.50	2303.80	0.44	0.6481

Note: *P value lower than 0.05; **The degree of freedom is *F*(2,82) in the main effect of the location of the ROI, *F*(1,46) in the main effect of the tissue characteristic, and *F*(2,82) in the interaction effect of LoR and TC. SS = sum of the square; MS = mean square. LoR = location of ROI; TC = tissue characteristic.

**Table 3:** Post-hoc pairwise comparisons analysis with Sidak adjustment in 0.073–0.198 Hz and 0.198–0.25 Hz.

Tumour	Frequency band (Hz)	Centre of tumour (c)	Periphery of tumour (p)	Normal muscle (m)	*P* value (p > c)	*P* value (p > m)	*P* value (c > m)
Malignant	0.073–0.198	20.46 ± 22.46	33.86 ± 32.16	16.88 ± 12.14	0.0049**	n.s.	n.s.
	0.198–0.25	61.38 ± 89.21	95.69 ± 110.20	38.85 ± 24.66	0.0127*	n.s.	n.s.
Benign	0.073–0.198	16.91 ± 10.89	32.62 ± 50.81	21.71 ± 23.19	n.s.	n.s.	n.s.
	0.198–0.25	41.20 ± 33.07	75.69 ± 125.20	44.32 ± 70.63	n.s.	n.s.	n.s.

Note: **P value lower than 0.05 after correction, which is a *P* value less than 0.0085. *P value lower than 0.1 after correction, which is a *P* value less than 0.0174. p > c: power of the periphery is higher than that of the centre. p > m: power of the periphery is higher than that of the normal muscle.

**Table 4:** Values of Intra-(M.J.W-1 and M.J.W-2) or Inter-(M.J.W-1–Z.J.Z/M.J.W-2–Z.J.Z) Rater Correlation Coefficient for the Reproducibility Analyses.

Frequency band (Hz)	ROIs in the centre of the tumour	ROIs in the periphery of the tumour	ROIs in the normal muscle
Band-1: 0.01–0.027	0.62^a^	0.68	0.80
	0.72^b^/0.59^c^	0.37/0.54	0.79/0.86
Band-2: 0.027–0.073	0.64	0.60	0.65
	0.80/0.71	0.59/0.64	0.45/0.66
Band-3: 0.073–0.198	0.81	0.49	0.58
	0.70/0.75	0.52/0.63	0.43/0.78
Band-4: 0.198–0.25	0.93	0.52	0.68
	0.81/0.83	0.63/0.64	0.66/0.87

Note: a = ICC values between M.J.W-1 and M.J.W-2; b = ICC values between M.J.W-1 and Z.J.Z; c = ICC values between M.J.W-2 and Z.J.Z.

## References

[b1] Dewhirst M. W. Concepts of oxygen transport at the microcirculatory level. Semin. Radiat. Oncol. 8, 143–150 (1998). 9634491 10.1016/s1053-4296(98)80040-4

[b2] Chaplin D. J., Olive P. L. & Durand R. E. Intermittent blood flow in a murine tumor: radiobiological effects. Cancer Res. 47, 597–601 (1987). 3791244

[b3] Rofstad E. K. & Maseide K. Radiobiological and immunohistochemical assessment of hypoxia in human melanoma xenografts: acute and chronic hypoxia in individual tumours. Int. J. Radiat. Biol. 75, 1377–1393 (1999). 10597912 10.1080/095530099139250

[b4] Durand R. E. Intermittent blood flow in solid tumours–an under-appreciated source of ‘drug resistance’. Cancer Metastasis Rev. 20, 57–61 (2001). 11831648 10.1023/a:1013181107707

[b5] Cairns R. A., Kalliomaki T. & Hill R. P. Acute (cyclic) hypoxia enhances spontaneous metastasis of KHT murine tumors. Cancer Res. 61, 8903–8908 (2001). 11751415

[b6] Intaglietta M., Myers R. R., Gross J. F. & Reinhold H. S. Dynamics of microvascular flow in implanted mouse mammary tumours. Bibl. Anat. 15, 273–276 (1977). 597155

[b7] Dewhirst M. W. *et al.* Microvascular studies on the origins of perfusion-limited hypoxia. Br. J. Cancer. 27, S247–S251 (1996). PMC2149984 8763890

[b8] Carmeliet P. & Jain R. K. Angiogenesis in cancer and other diseases. Nature. 407, 249–257 (2000). 11001068 10.1038/35025220

[b9] Dewhirst M. W., Braun R. D. & Lanzen J. L. Temporal changes in pO2 of R3230AC tumors in fisher-344 rats. Int. J. Radiat. Oncol. 42, 723–726 (1998). 10.1016/s0360-3016(98)00304-6 9845084

[b10] Dewhirst M. W., Secomb T. W., Ong E. T., Hsu R. & Gross J. F. Determination of local oxygen consumption rates in tumors. Cancer Res. 54, 3333–3336 (1994). 8012945

[b11] Brurberg K. J., Graff B. A. & Rofstad E. K. Temporal heterogeneity in oxygen tension in human melanoma xenografts. Br. J. Cancer. 89, 350–356 (2003). 12865929 10.1038/sj.bjc.6601047 PMC2394245

[b12] Brurberg K. G., Graff B. A., Olsen D. R. & Rofstad E. K. Tumor-line specific pO2 fluctuations in human melanoma xenograft. Int. J. Radiat. Oncol. Biol. Phys. 58, 403–409 (2004). 10.1016/j.ijrobp.2003.09.049 14751509

[b13] Dewhirst M. W. *et al.* Quantification of longitudinal tissue pO2 gradients in window chamber tumours: impact on tumour hypoxia. Br. J. Cancer. 79,1717–1722 (1999). 10206282 10.1038/sj.bjc.6690273 PMC2362789

[b14] Wang K., Yorke E., Nehmeh S. A., Humm J. L. & Ling C. C. Modeling acute and chronic hypoxia using serial images of 18F-FMISO PET. Med. Phys. 36, 4400–4408 (2009). 19928070 10.1118/1.3213092 PMC2852451

[b15] Magat J., Jordan B. F., Cron G. O. & Gallez B. Noninvasive mapping of spontaneous fluctuations in tumor oxygenation using 19F MRI. Med. Phys. 37, 5434–5441 (2010). 21089779 10.1118/1.3484056

[b16] Ogawa S., Lee T. M., Kay A. R. & Tank D. W. Brain magnetic resonance imaging with contrast dependent on blood oxygenation. Proc. Natl. Acad. Sci. 87, 9868–9872 (1990). 2124706 10.1073/pnas.87.24.9868 PMC55275

[b17] Howe F. A., Robinson S. P., McIntyre D. J., Stubbs M. & Griffiths J. R. Issues in flow and oxygenation dependent contrast (FLOOD) imaging of tumours. NMR Biomed. 14, 497–506 (2001). 11746943 10.1002/nbm.716

[b18] Baudelet C. & Gallez B. Current Issues in the Utility of Blood Oxygen Level Dependent MRI for the Assessment of Modulations in Tumor Oxygenation. Curr. Med. Imaging Rev. 3, 229–243 (2005).

[b19] Baudelet C. *et al.* Physiological noise in murine solid tumours using T2*-weighted gradient-echo imaging: a marker of tumour acute hypoxia? Phys. Med. Biol. 49, 3389–3411 (2004). 15379021 10.1088/0031-9155/49/15/006

[b20] Baudelet C. *et al.* The role of vessel maturation and vessel functionality in spontaneous fluctuations of T2*-weighted GRE signal within tumors. NMR Biomed. 19, 69–76 (2006). 16411170 10.1002/nbm.1002

[b21] Gonçalves M. R. *et al.* Decomposition of spontaneous fluctuations in tumour oxygenation using BOLD MRI and independent component analysis. Br. J. Cancer. 113, 1168–1177 (2015). 26484634 10.1038/bjc.2015.270 PMC4647875

[b22] Buzsaki G. & Draguhn A. Neuronal oscillations in cortical networks. Science. 304, 1926–1929 (2004). 15218136 10.1126/science.1099745

[b23] Ogawa S. *et al.* Functional brain mapping by blood oxygenation level-dependent contrast magnetic resonance imaging. A comparison of signal characteristics with a biophysical model. Biophys. J. 64, 803–812 (1993). 8386018 10.1016/S0006-3495(93)81441-3 PMC1262394

[b24] Baudelet C. & Gallez B. Effect of anesthesia on the signal intensity in tumors using BOLD-MRI: comparison with flow measurements by Laser Doppler flowmetry and oxygen measurements by luminescence-based probes. Magn. Reson. Imaging. 22, 905–912 (2004). 15288130 10.1016/j.mri.2004.02.005

[b25] Viviani R., Messina I. & Walter M. Resting state functional connectivity in perfusion imaging: correlation maps with BOLD connectivity and resting state perfusion. PloS One. 6, e27050 (2011). 22073252 10.1371/journal.pone.0027050 PMC3208575

[b26] Bruyns-Haylett M. *et al.* The resting-state neurovascular coupling relationship: rapid changes in spontaneous neural activity in the somatosensory cortex are associated with haemodynamic fluctuations that resemble stimulus-evoked haemodynamics. Eur. J. Neurosci. 38, 2902–2916 (2013). 23841797 10.1111/ejn.12295

[b27] Häfner H. M. *et al.* Wavelet analysis of cutaneous blood flow in melanocytic skin lesions. J. Vasc. Res. 42, 38–46 (2005). 15637439 10.1159/000082975

[b28] Meyer J. U., Borgstrom P., Lindbom L. & Intaglietta M. Vasomotion patterns in skeletal muscle arterioles during changes in arterial pressure. Microvasc. Res. 35, 193–203 (1988). 3367792 10.1016/0026-2862(88)90062-3

[b29] Delgado E., Marques-Neves C., Rocha I., Sales-Luís J. & Silva-Carvalho L. Myogenic oscillations in rabbit ocular vasculature are very low frequency. Ophthalmic Res. 50, 123–128 (2013). 23899812 10.1159/000351629

[b30] Yan C. G. & Zang Y. F. DPARSF: A MATLAB Toolbox for “Pipeline” Data Analysis of Resting-State fMRI. Front. Syst. Neurosci. 4, 13 (2010). 20577591 10.3389/fnsys.2010.00013 PMC2889691

[b31] Song X. W. *et al.* REST: a toolkit for resting-state functional magnetic resonance imaging data processing. PloS One. 6, e25031 (2011). 21949842 10.1371/journal.pone.0025031 PMC3176805

[b32] Simonsen T. G., Gaustad J. V., Leinaas M. N. & Rofstad E. K. High interstitial fluid pressure is associated with tumor-line specific vascular abnormalities in human melanoma xenografts. PloS One. 7, e40006 (2012). 22768196 10.1371/journal.pone.0040006 PMC3386940

[b33] Zang Y. F. *et al.* Altered baseline brain activity in children with ADHD revealed by resting-state functional MRI. Brain Dev. 29, 83–91 (2007). 16919409 10.1016/j.braindev.2006.07.002

[b34] Zhang H. *et al.* Test-retest assessment of independent component analysis-derived resting-state functional connectivity based on functional near-infrared spectroscopy. NeuroImage. 55, 607–615 (2011). 21146616 10.1016/j.neuroimage.2010.12.007

